# Armored probiotics for oral delivery

**DOI:** 10.1002/SMMD.20230019

**Published:** 2023-07-20

**Authors:** Xinyuan Yang, Chong Wang, Qiao Wang, Zhuohao Zhang, Weimin Nie, Luoran Shang

**Affiliations:** ^1^ Zhongshan‐Xuhui Hospital and the Shanghai Key Laboratory of Medical Epigenetics, the International Co‐laboratory of Medical Epigenetics and Metabolism (Ministry of Science and Technology) Institutes of Biomedical Sciences Fudan University Shanghai China; ^2^ Key Laboratory of Smart Drug Delivery School of Pharmacy Fudan University Shanghai China

**Keywords:** encapsulation, functional biomaterials, gastrointestinal environment, oral delivery, probiotics, surface coating

## Abstract

As a kind of intestinal flora regulator, probiotics show great potential in the treatment of many diseases. However, orally delivered probiotics are often vulnerable to unfriendly gastrointestinal environments, resulting in a low survival rate and decreased therapeutic efficacy. Decorating or encapsulating probiotics with functional biomaterials has become a facile yet useful strategy, and probiotics can be given different functions by wearing different armors. This review systematically discusses the challenges faced by oral probiotics and the research progress of armored probiotics delivery systems. We focus on how various functional armors help probiotics overcome different obstacles and achieve efficient delivery. We also introduce the applications of armor probiotics in disease treatment and analyze the future trends of developing advanced probiotics‐based therapies.

## Graphical abstract

In this review, we systematically discuss the challenges faced by oral probiotics and the research progress of armored probiotics delivery systems. We focus on how various functional armors help probiotics overcome different obstacles and achieve efficient delivery. We also introduce the applications of armor probiotics in disease treatment and analyze the future trends of developing advanced probiotics‐based therapies.

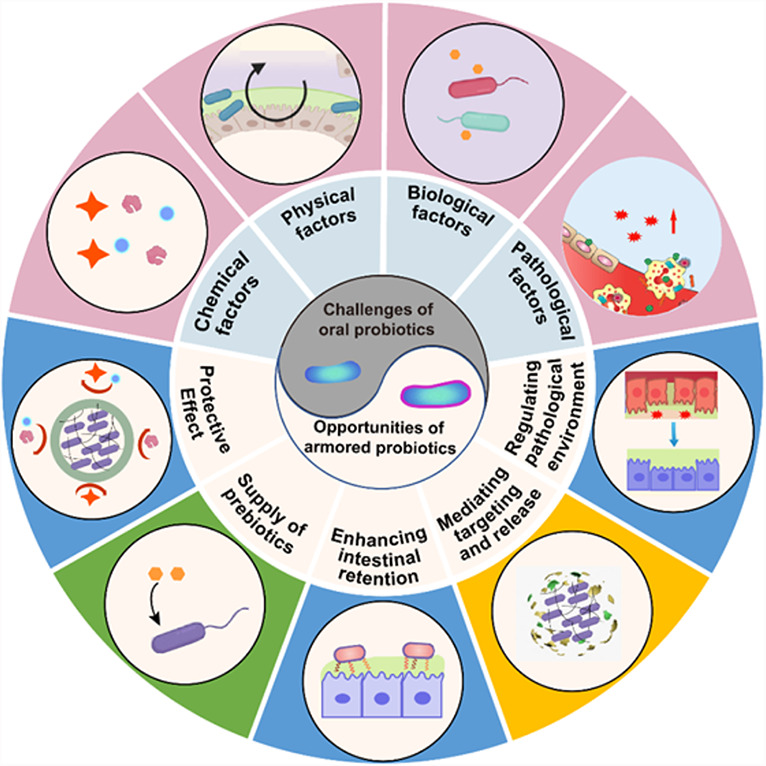

**Key points**

- Oral probiotics are a promising approach for disease treatment due to their ability to modulate the gut microbiota.
- By equipping probiotics with different functional materials (armor), probiotics can be endowed with functions to resist adverse challenges when navigating the gastrointestinal tract.
- It is expected to promote a deep cross‐fusion of material science and microbiological science and provide new ideas for the further application of oral probiotics in the treatment and prevention of diseases.

## 1 INTRODUCTION

The human intestinal tract is inhabited by trillions of microorganisms, which compose a complex and dynamic ecosystem.^1^ The gut microbiota co‐evolves with its host, forming a symbiotic relationship.^2^ As the knowledge of gut microbiota increases, growing evidence has demonstrated that the balance of gut microbiota is critical for maintaining intestinal homeostasis and host health.^3^ ^,^ ^4^ In the healthy state, commensal intestinal microbiota maintain homeostasis by regulating physiological processes. However, some internal or external factors (diet, antibiotics, etc.) can alter the commensal microbial community, leading to the disturbance of intestinal homeostasis.^5^ ^,^ ^6^ The state of dysbiosis can lead to various diseases, such as inflammatory bowel disease (IBD),^7^ ^,^ ^8^ obesity,^9^ diabetes,^10^ irritable bowel syndrome^11^ and cancer,^12^ etc. Probiotics are commonly defined as “live microorganisms that are beneficial to the host when administered in sufficient quantities”.^13^ ^,^ ^14^ The introduced probiotics can exert broad beneficial effects,^15^ including regulating the host immune system, improving the intestinal barrier function, inhibiting the colonization of pathogens, and regulating the ecological balance of the intestinal flora.^16^ Therefore, probiotics have been widely used to prevent and cure diseases.

Oral delivery of probiotics is a simple and widely used means.^17^ However, the complex physiological condition and pathological environment of the gastrointestinal tract severely limit the survival and colonization of oral probiotics in the gut. After oral administration, probiotics undergo gastric acid, bile salts, and digestive enzymes, which can inactivate most probiotics.^18^ ^,^ ^19^ Besides, the peristalsis and rapid transit of the gastrointestinal tract will accelerate the removal of probiotics from the gut, reducing their adhesion and growth in the intestine.^20^ In addition, significantly elevated reactive oxygen species (ROS) levels in the inflammatory intestinal diseases microenvironment can severely damage the cell walls of probiotics, leading to the death of probiotics.^21^, ^22^, ^23^, ^24^ Moreover, abnormally proliferating pathogenic bacteria can compete for nutrients and niches with probiotics, and affect the colonization of probiotics in the intestinal tract.^25^, ^26^, ^27^, ^28^, ^29^ These challenges seriously hinder the therapeutic effects of orally delivered probiotics. Therefore, strategies need to be developed to protect probiotics during gastrointestinal transit and enhance their colonization, thus improving the bioavailability and therapeutic efficacy.

Biomaterials have been prevalently utilized as functional armors to decorate or encapsulate probiotics for enhancing their survival and colonization. Different biomaterials have been employed to endow probiotics with different functions. For example, some polysaccharides, proteins, or lipid films have been explored to make probiotics resistant to gastric acid or enzymes and improve their survival rate.^30^, ^31^, ^32^, ^33^ Mucoadhesive polymers or bacterial biofilms can increase the adhesion of probiotics to the intestinal mucosa and improve their retention time in the intestinal tract.^34^ ^,^ ^35^ The use of prebiotics could promote the proliferation of probiotics in the intestines.^36^ ^,^ ^37^ More intriguingly, with the optimal design of the probiotics‐biomaterials composite system, the probiotics could be endowed with the abilities to respond to, target, resist or modulate the pathological environment of intestinal diseases.^38^, ^39^, ^40^, ^41^, ^42^, ^43^, ^44^

Based on these achievements, this review summarizes the research advances in armored probiotics for oral delivery. Although there are other excellent reviews covering this subject, a majority of them emphasize the formation techniques of armored/encapsulated probiotics. Alternatively, this review focuses on the functions that materials confer on probiotics. Specifically, we first introduce the environmental characteristics of the gastrointestinal tract and the obstacles probiotics face during oral delivery. We then discuss how armored probiotics have gained various functions to overcome these obstacles. After that, we shift to the main applications of armored probiotics for disease treatments. Finally, we provide critical thinking about remaining challenges and future trends in oral delivery of probiotics.

## 2 ACTION MECHANISMS OF PROBIOTICS

**FIGURE 1:**
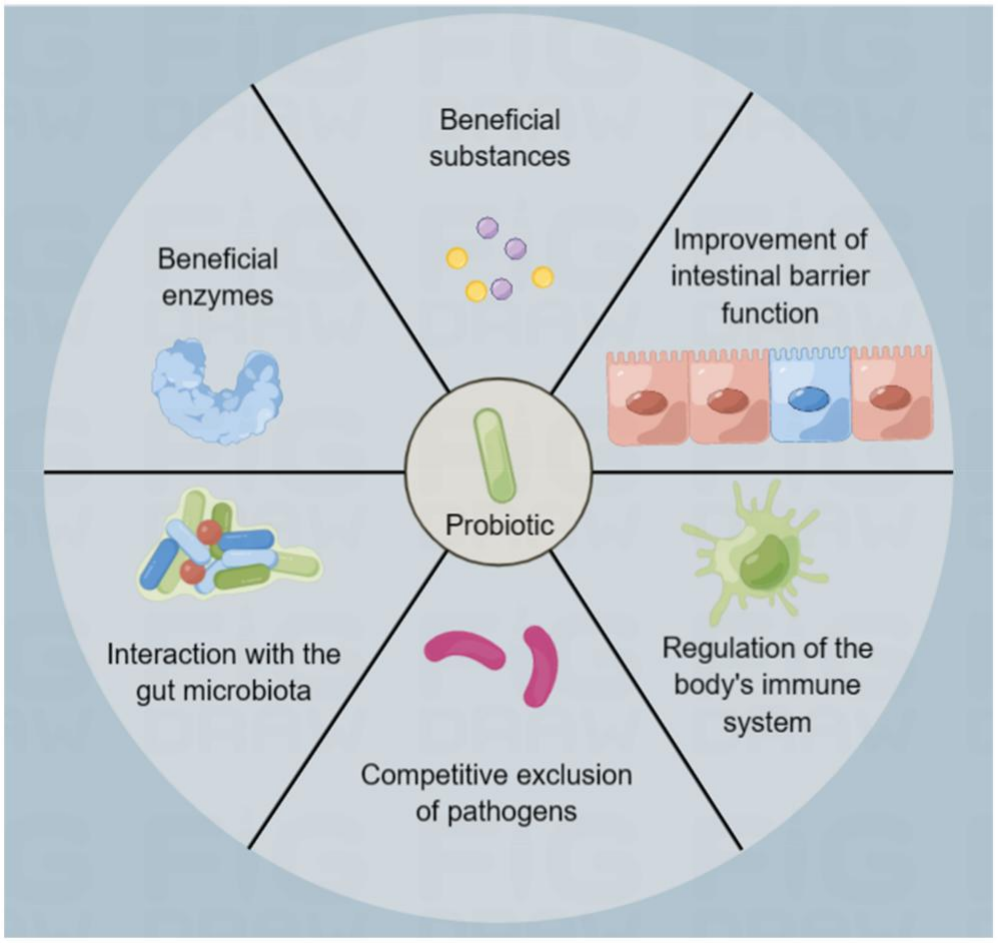
A variety of mechanisms that may contribute to probiotics' beneficial effects on host health, such as the production of beneficial enzymes and other substances, improvement of intestinal barrier function, direct interaction with the gut microbiota, competitive exclusion of pathogens, and regulation of the immune system. This figure was drawn by Figdraw.

As an active microbial supplement, the criteria for probiotics mainly include being alive in the gastrointestinal tract, non‐pathogenicity, producing beneficial substances, and demonstrating immune modulating capabilities.^45^ There are many types of probiotics, in which *Bifidobacterium* and *Lactobacillus* are two of the most broadly used genera in research.^46^ In addition, *Enterococcus*, *Saccharomyces*, and *Escherichia coli* Nissle 1917 (EcN) are also widely used.^15^ The latest advances in microbiology have demonstrated the importance of intestinal probiotics in regulating and maintaining human health.^16^ Here, we summarize several major beneficial effects probiotics exert on the human body (Figure 1), each of which is supported by representative examples:

### 2.1 Production of beneficial enzymes

*Streptococcus salivarius* subsp. *thermophilus* can secrete β‐galactosidase in the gastrointestinal tract to decompose lactose into digestible galactose and glucose, thereby effectively alleviating lactose intolerance.^47^ Besides, *Lactobacillus plantarum* ECGC 13110402 shows high activity in hydrolyzing bile salts, and its potential in lowering the level of cholesterol in hypercholesterolemic adults has been confirmed.^48^

### 2.2 Production of other beneficial substances

In addition to the above‐mentioned enzymes, *Lactobacillus rhamnosus* GG can produce soluble proteins that prevent cytokine‐induced epithelial damage and apoptosis.^49^ *Lactobacillus paracasei* NFRI 7415 can produce neurotransmitter gamma‐aminobutyric acid, which is known to affect brain function.^50^

### 2.3 Improvement of intestinal barrier functions

EcN has been demonstrated to increase the expression of multiple MUC genes in intestinal cells.^51^, ^52^, ^53^ By stimulating the secretion of mucin, EcN can help maintain the intestinal barrier integrity and functions. Mattar et al. proved that *Lactobacillus casei* strain GG could increase the expression of the MUC2 gene in a Caco‐2 cell culture model.^54^ Also, in the Caco‐2 cell model, *Lactobacillus plantarum* MB452 has been validated to upregulate the expression of tight junction‐associated genes, which play key roles in intestinal barrier functions.^55^ ^,^ ^56^

### 2.4 Interaction with the gut microbiota

Probiotics can exert their efficacy by interacting with other microorganisms in the gut. Vuyst and colleagues proved that *Bifidobacterium longum* subsp. *longum* NCC2705 can ferment carbohydrates to produce acetate and cross‐feed other colonic bacteria to improve their growth and proliferation.^57^

### 2.5 Competitive exclusion of pathogens

A study by Raffatellu and colleagues showed that EcN can reduce the colonization of Enterobacteriaceae and *Salmonella typhimurium* in the inflamed intestine, through the production of microcin.^58^ *Bifidobacterium longum* subsp. *longum* JCM 1217^T^ can metabolize carbohydrates into acetate and inhibit the growth and colonization of pathogenic bacteria.^59^ ^,^ ^60^

### 2.6 Regulation of the immune system

Probiotics also have certain effects on regulating the host immune system. Mizuno and colleagues proved that *Lactococcus lactis* subsp. *cremoris* FC can downregulate the pro‐inflammatory IL‐8 mRNA expression at the cellular level and inhibit the nuclear translocation of NF‐κB in RAW264.7 cells to relieve intestinal inflammation.^61^

## 3 CHALLENGES IN ORAL DELIVERY OF PROBIOTICS

**FIGURE 2:**
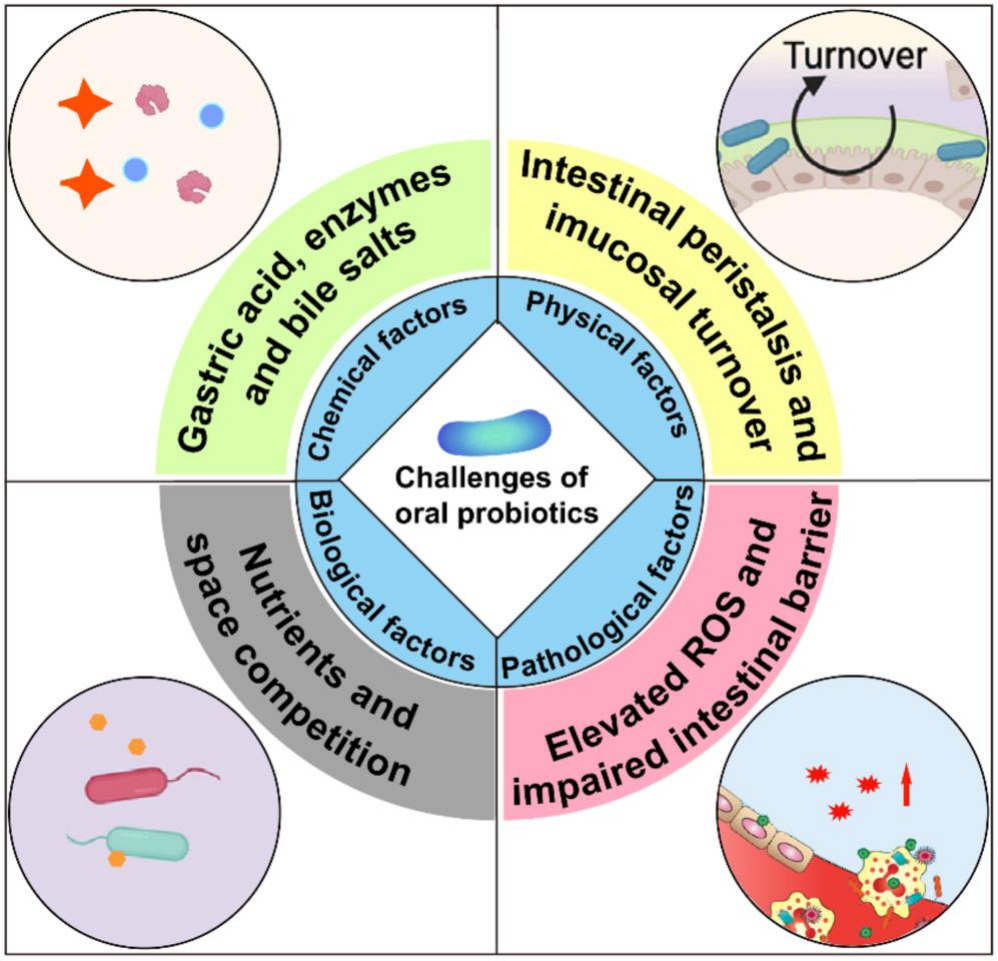
Challenges in oral delivery of probiotics. Reproduced with permission.^62^ Copyright 2022, Elsevier. Reproduced under terms of the CC‐BY license.^63^ Copyright 2019, The Authors, published by Springer Nature. Oral probiotics will encounter various obstacles, including chemical factors such as gastric acid, bile salts and enzymes; physical factors such as intestinal peristalsis and mucosal turnover; biological factors, that is, intestinal native microorganisms; and pathological factors such as elevated reactive oxygen species (ROS) and impaired intestinal barrier.

Delivery of probiotics is a promising approach for disease prevention and treatment due to their great benefits to human health. Generally, oral delivery is the simplest and most commonly used route of delivering probiotics. During this process, the probiotics are introduced in an active form and in sufficient amounts, and they need to overcome several obstacles in the gastrointestinal tract (gastric acid, enzymes, bile salts, peristalsis, etc). Probiotics also need to be able to compete for resources in the gastrointestinal tract, promoting their own growth and persistence. When replication is equal to or exceeds the washout of the gut, the introduced probiotics can be considered successfully colonizing in the gut.^27^ However, to date, maintaining the activity of oral probiotics in complex gastrointestinal conditions remains a formidable challenge. During the transit through the stomach and small intestine to reach the colon, live probiotics will encounter a variety of harsh environments, in which multiple factors can affect their survival and colonization (Figure 2).

### 3.1 Chemical factors

Probiotics can be inactivated by gastric acid in gastric juice and digestive enzymes (lipase and protease) as well as bile salts in intestinal juice.^17^ The large amount of H^+^ in gastric acid may change the zeta potential of ingested probiotics, which affects the permeability of their membrane and results in deactivation.^35^, ^64^ Besides, high concentrations of bile can dissolve the lipids of the probiotic membrane, causing leakage and membrane disruption. Moreover, digestive enzymes can also cause degradation and loss of viability of probiotics.^65^, ^66^, ^67^

### 3.2 Physical factors

The peristalsis in the gastrointestinal tract, that is, muscle contractions associated with the breakdown and transport of food, leads to rapid transit times, which will limit the retention of probiotics in the gut, reducing their adhesion and growth.^68^ ^,^ ^69^ Turnover in epithelial cells and mucus along the gastrointestinal tract can also remove probiotics out of the gastrointestinal tract and affect their residence time.^70^ ^,^ ^62^

### 3.3 Biological factors

When ingested bacteria reach the gut, they often fail to gain a competitive advantage over native microbiota, resulting in their competitive exclusion, which is referred to as “colonization resistance”.^62^, ^71^ Oral ingested probiotics will also face this problem. The uptake of nutrients and the availability of living space are essential conditions for the gut colonization of exotic probiotics.^72^ ^,^ ^73^ Therefore, the competition of resources and living spaces and the interactions with the resident intestinal flora will largely influence the success of the colonization of probiotics.^27^

### 3.4 Pathological factors

The pathological microenvironment of intestinal diseases can seriously affect the survival and colonization of probiotics in the intestine. For example, in the pathological microenvironment of IBD, Numerous immune cells (such as macrophages, neutrophils, leukocytes, etc.) are extensively infiltrated, leading to a significant increase in ROS levels at the lesion site.^74^, ^75^, ^76^, ^77^ Excessive ROS has a powerful oxidative effect on lipids and proteins on the cell membrane of probiotics, which can seriously damage the probiotics' cell membrane and lead to the inactivation of probiotics.^23^ ^,^ ^78^ ^,^ ^79^ In addition, the mucus layer is essential for maintaining the mucosal barrier of the gastrointestinal (GI) tract and is also a habitat for probiotic growth, colonization, and reproduction^80^ ^,^ ^81^. However, the mucus layer in the lesion site of IBD is severely damaged and the intestinal permeability is significantly increased.^82^ ^,^ ^83^ In that case, orally delivered probiotics may not colonize the colon site. On the contrary, they may enter the blood and transfer to other organs, causing serious side effects.^84^ What's more, over‐proliferating pathogens at the lesion site also compete with probiotics for nutrients and ecological niches,^29^ ^,^ ^85^ ^,^ ^86^ preventing probiotics from colonizing the gut.

## 4 STRATEGIES FOR ARMORING PROBIOTICS

Armoring probiotics with biomaterials or functional motifs can significantly improve their viability and confer more functionalities on them for enhanced therapeutic efficacy. With the in‐depth development of research, a large number of functional biomaterials or motifs as well as encapsulation means have been used to help probiotics overcome the challenges and achieve effective oral administration.^87^ Extensive studies have demonstrated that appropriate armors on probiotics can have a powerful potential to enhance their survival and colonization, and thus leading to better therapeutic effects. Specifically, based on the different challenges to be tackled, we here categorize the strategies for armoring probiotics as follows:

### 4.1 Protection from the harsh GI environment

**FIGURE 3:**
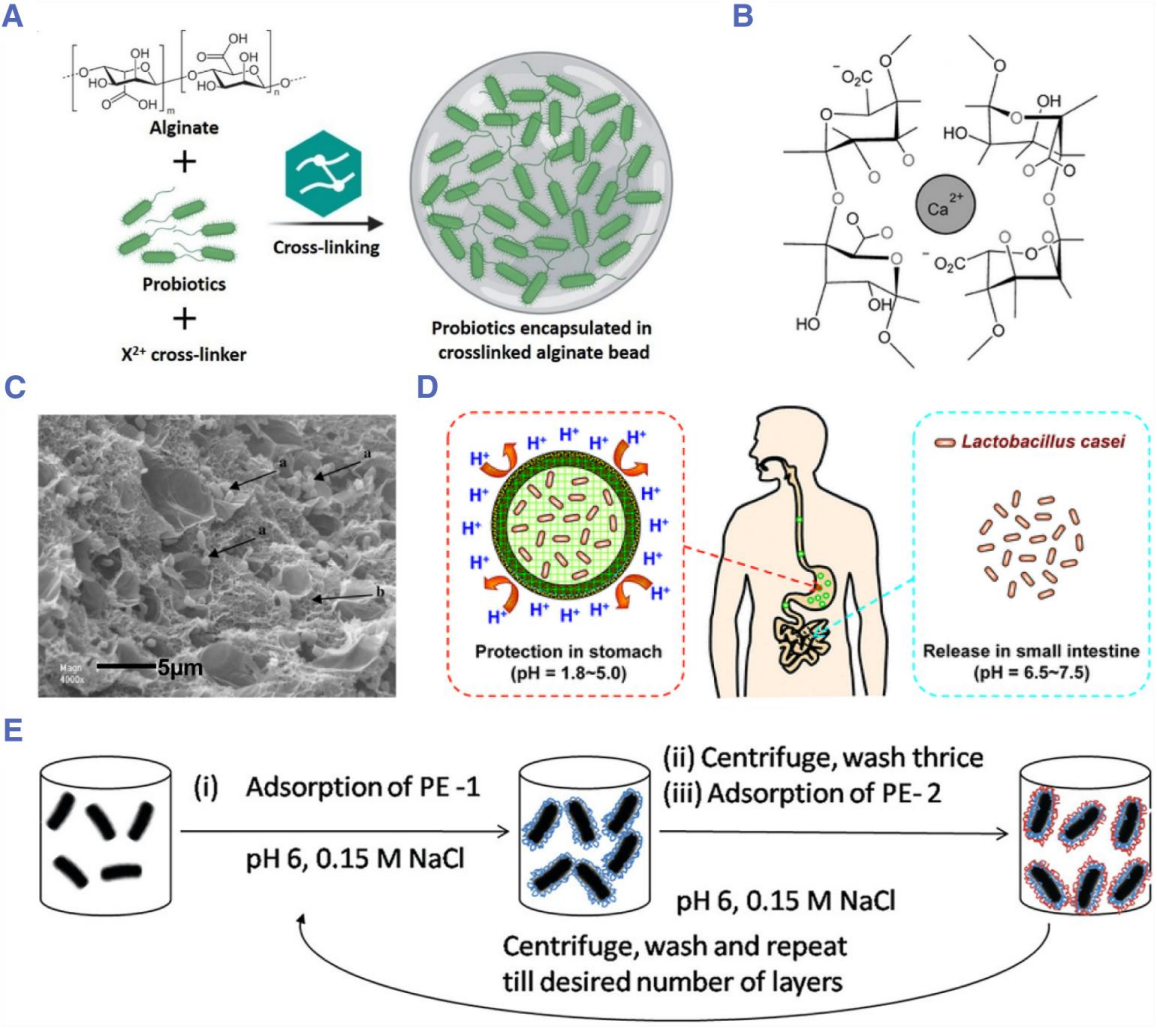
(A) Probiotics encapsulated in ionic‐crosslinked alginate beads. Reproduced with permission.^92^ Copyright 2020, Elsevier. (B) “Egg‐box” structure of calcium alginate. Reproduced with permission.^93^ Copyright 2022, Elsevier. (C) Cross‐section of hydrogel beads encapsulated with *Lactobacillus plantarum* spp. The black arrow annotates *L. plantarum* spp. Scale bar: 5 µm. Reproduced with permission.^94^ Copyright 2009, Elsevier. (D) Alginate/protamine beads offer improved protection for *Lactobacillus casei* in stomach and allow for rapid release of *L. casei* in the small intestine. Reproduced with permission.^95^ Copyright 2014, American Chemical Society. (E) LBL assembly of polyelectrolytes on the cell wall of probiotics. Reproduced with permission.^96^ Copyright 2011, American Chemical Society.

The first challenge after oral delivery of probiotics is the severe physiological environment of the gastrointestinal tract. Microencapsulation or surface modification of probiotics is an effective means to protect them from inactivation.^88^ Polysaccharides are among the most commonly used “armor” materials for probiotic encapsulation.^89^ ^,^ ^90^ For example, Alginate has been widely studied for the encapsulation of probiotics due to their good biocompatibility, gel‐forming ability via ionic crosslinking, and pH‐responsive property.^91^ Alginate can form an “egg‐box”‐structured gel network with some metal ions (such as calcium ions, zinc ions, copper ions, manganese ions, and so on). Accordingly, alginate beads have been proposed to effectively encapsulate probiotics and provide a protective effect (Figure 3A–C).^97^ ^,^ ^98^ Under acidic conditions, alginate shrinks and forms insoluble algal skin‐like structures,^91^ which may limit gastric juice diffusion into bacterial cells, thereby improving the survival of probiotics to some extent.^99^

Since the calcium alginate hydrogel has a high porosity, it has been used in combination with other materials to improve the protective effect on probiotics against the harsh environments.^93^, ^94^, ^100^, ^101^ For example, by introducing protamine into sodium alginate, Chu and colleagues prepared beads with an alginate/protamine shell to encapsulate *Lactobacillus casei* with an increased survival rate of about 60 times compared to conventional sodium alginate beads.^95^ When in the stomach, the diffusion channel of the shell is blocked, which prevents the gastric juice from entering the interior of the beads. After transiting into the small intestine, due to the dual action of trypsin and neutral pH, the bead dissolved rapidly to release the probiotics (Figure 3D). Chen and colleagues used positively charged chitosan and negatively charged alginate to coat EcN via a layer‐by‐layer (LBL) electrostatic self‐assembly strategy.^102^ Such chitosan/sodium alginate coating showed better protection of EcN compared to the clinically used enteric coating material (Eudragit L100‐55). This may be attributed to the insoluble skin‐like structures formed by alginate. In addition to alginate‐based coatings, Raichur and colleagues encapsulated *Lactobacillus acidophilus* by LBL coating technology with carboxymethyl cellulose and chitosan to enhance their vitality in the gastrointestinal tract, as shown in Figure 3E.^96^

**FIGURE 4:**
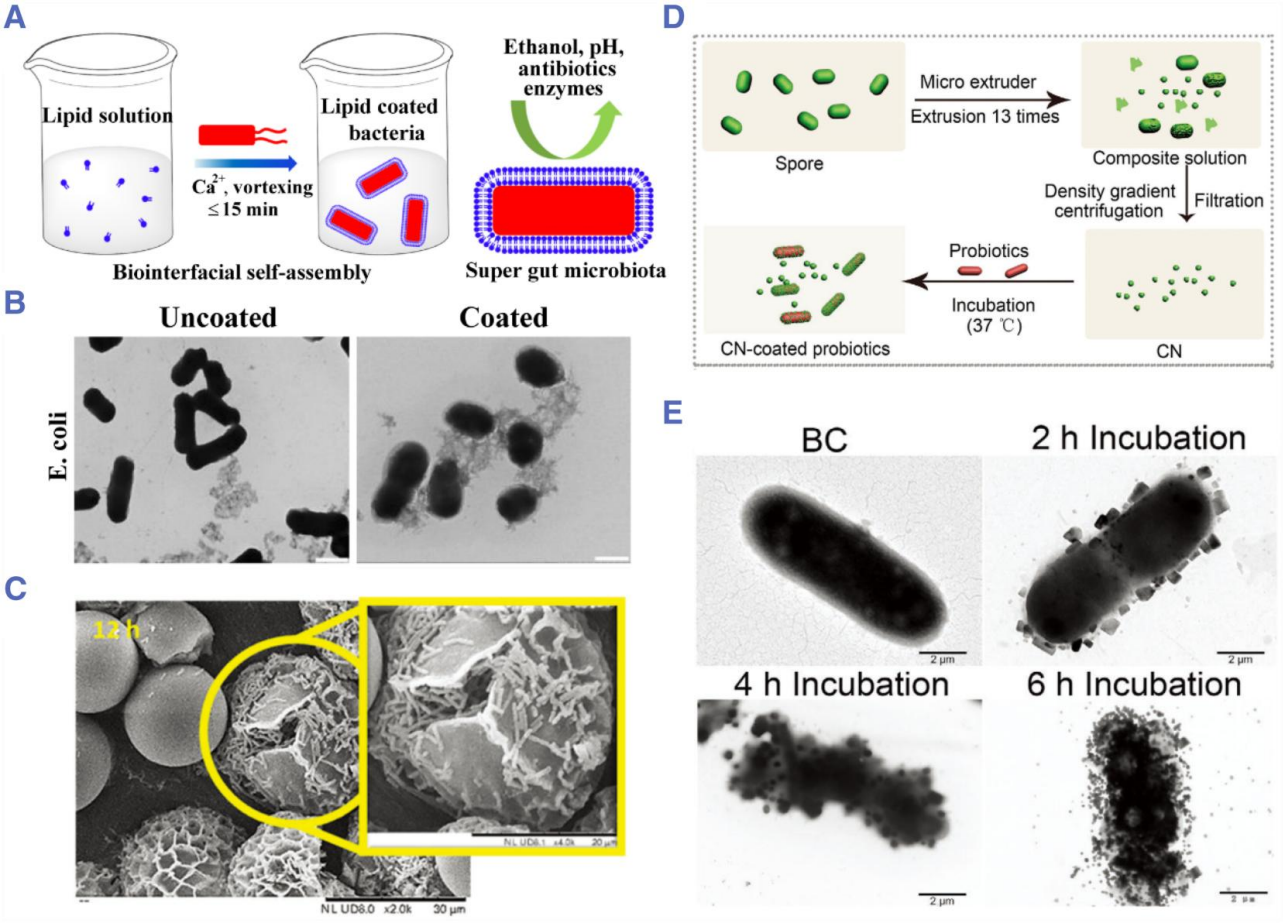
(A) Lipid membrane‐coated probiotics by biointerfacial self‐assembly. (B) Transmission electron microscope (TEM) images of uncoated probiotics and coated probiotics. Scale bars: 1 µm. Reproduced under terms of the CC‐BY license.^32^ Copyright 2019, The Authors, published by Springer Nature. (C) Scanning electron microscope (SEM) images of encapsulated *Lactobacillus casei* after incubation at 37°C for 12 h. Reproduced with permission.^103^ Copyright 2021, John Wiley and Sons. (D) Preparation of the spore‐derived nanoparticles and the coating of probiotics. CN refers to coat nanoparticles. (E) TEM images of CN‐coated probiotics during the preparation. BC refers to *Bacillus coagulans*. Scale bars: 2 µm. Reproduced with permission.^104^ Copyright 2021, John Wiley and Sons.

Apart from polysaccharides, various other types of materials have been explored as probiotic coatings. For example, Liu and colleagues proposed encapsulating probiotics with lipid membranes through biointerfacial supramolecular self‐assembly, which significantly improved the survival rate of probiotics in various extreme conditions (Figure 4A,B).^32^ Notably, sporopollenin exine capsules, the outer shells of pollen, can be used as an alternative encapsulant for probiotics due to their good mechanical properties and stress resistance.^105^ ^,^ ^106^ Konteles and colleagues used sporopollenin exine capsules to encapsulate *Lactobacillus casei* and demonstrated that the encapsulated probiotics exhibited higher viability in all simulated mediums compared to free probiotics.^103^ Besides, as probiotics proliferated within the capsules, the capsules will be ruptured and the live probiotics can be released (Figure 4C). Another intriguing material is spores. Inspired by the strong resistance of probiotic spores in the stomach, Wang and colleagues transformed the spores into multifunctional nanoparticles by mechanical extrusion, and then coated these nanoparticles on the surface of probiotics, thereby protecting probiotics from harsh stomach conditions (Figure 4D,E).^104^ Overall, the various coating or encapsulation strategies can provide physical or chemical barriers for probiotics to effectively resist the harsh physiological environments in the GI tract.

### 4.2 Supply of prebiotics

Prebiotics are generally defined as substrates selectively exploited by host microorganisms presenting health benefits.^107^ Prebiotics are primarily carbohydrate‐based, but research also indicate other substances such as polyphenols and polyunsaturated fatty acids exert prebiotic effects.^108^ ^,^ ^109^ Prebiotics have powerful potential to promote the bio‐metabolism, viability and growth of probiotics.^110^ ^,^ ^111^ Additionally, prebiotics can increase the survival rate of probiotics passing through the upper gastrointestinal tract, thereby enhancing their bioeffects in the large intestine.^112^, ^113^, ^114^ Therefore, co‐delivery of probiotics and prebiotics is also commonly used for enhancing the oral delivery efficacy of probiotics. For example, Subirade and colleagues demonstrated that the survival of encapsulated probiotics under acidic conditions in vitro was significantly improved with the addition of inulin as a co‐encapsulant.^115^ Okuro and colleagues co‐encapsulated *Lactobacillus acidophilus* with prebiotics (inulin or polydextrose) via a spray chilling process, which greatly enhanced the viability of *L. acidophilus* in simulated gastric and intestinal fluids.^116^ In addition, the co‐encapsulation of *Eleutherine americana* oligosaccharide extract and *Lactobacillus* TISTR1465 not only improves the vitality of probiotics but also significantly enhances its ability in resisting pathogenic bacteria.^117^

**FIGURE 5:**
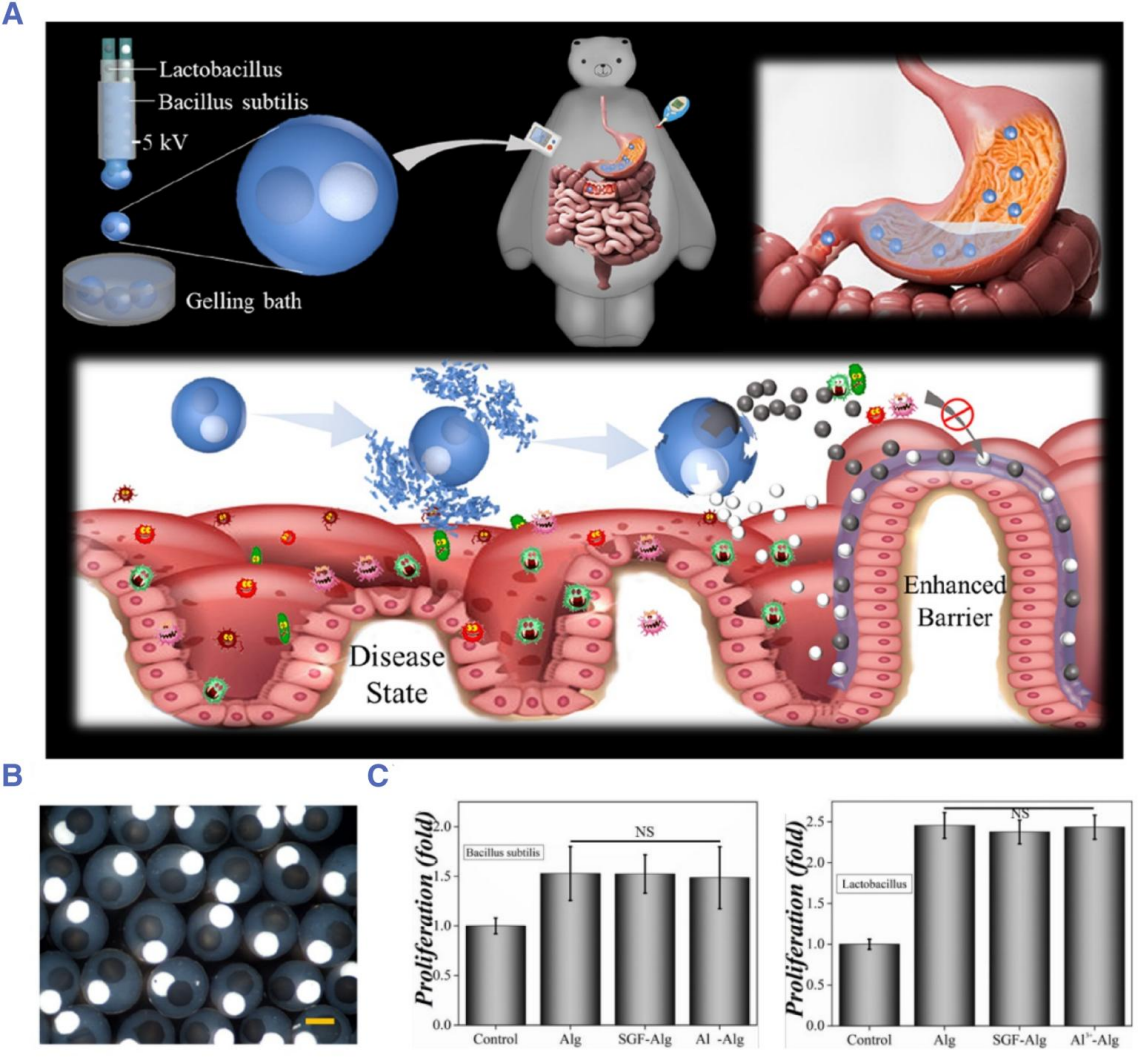
(A) Schematic of the double‐core prebiotic microcapsules containing two compartments encapsulating two kinds of probiotics and the strategy of synergistic delivery of two probiotics. (B) Bright‐field microscopic images of the double‐core microcapsules. Scale bar: 100 µm. (C) Proliferation of *Bacillus Subtilis* and *Lactobacillus* cultured with alginate, SGF‐treated alginate, and Al^3+^‐alginate for 24 h. Reproduced with permission.^118^ Copyright 2020, American Chemical Society.

Recent studies have also explored carriers with novel configurations for co‐encapsulating probiotics and prebiotics. As an example, Shang and colleagues encapsulated *Lactobacillus* and *Bacillus subtilis* into calcium alginate dual‐core microcapsules with separate compartments by electrostatically driven microfluidics (Figure 5A,B).^118^ By creating separate compartments, the two probiotics can mutually promote without affecting each other. Besides, because the degradation product of alginate is prebiotics, it can promote the proliferation of probiotics, so as to realize the synergistic effect between two different probiotics and between prebiotics and probiotics (Figure 5C). In addition, Zhang and colleagues prepared prebiotic‐encapsulated probiotic spores for oral delivery through host‐guest chemistry between *Clostridium butyricum* and dextran.^37^ Dextran not only promoted the reproduction of *Clostridium butyricum* but also produced a large amount of short‐chain fatty acids (SCFAs) upon the fermentation of *Clostridium butyricum*, which had a significant anti‐tumor effect.

### 4.3 Enhancing intestinal retention

**FIGURE 6:**
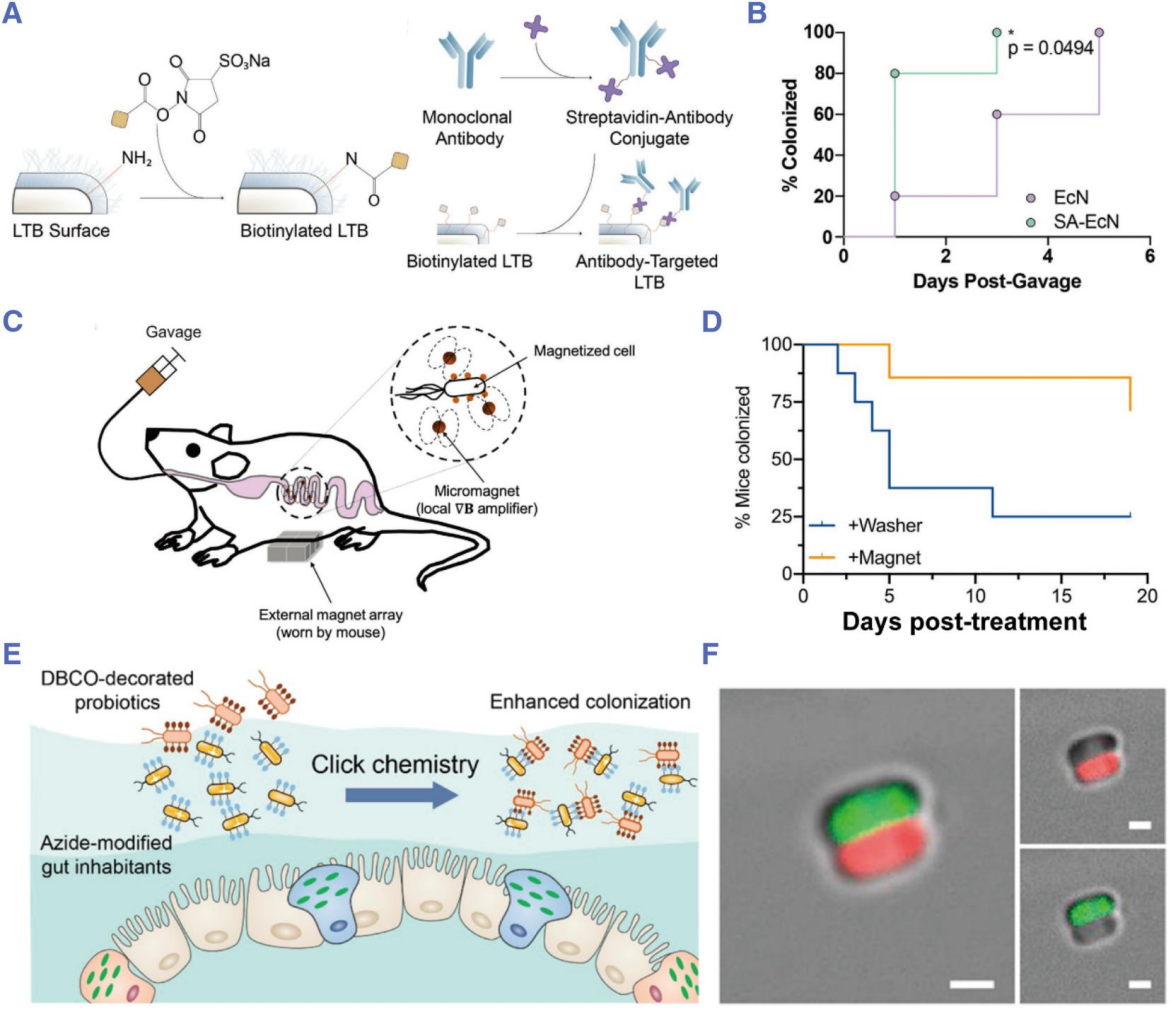
(A) Antibodies attachment to the surface of probiotics via biotin‐streptavidin reaction. (B) Kinetics of probiotic colonization. SA refers to synthetic adhesins. Reproduced with permission.^120^ Copyright 2020, John Wiley and Sons. (C) Orally administered magnetized probiotic cells captured by external magnets. (D) Percentage of mice with EcN detectable in the feces. Reproduced with permission.^121^ Copyright 2021, John Wiley and Sons. (E) Schematic illustration of bioorthogonal conjugation‐mediated enhancement of probiotic colonization in the gut. (F) Confocal laser scanning microscope (CLSM) images of bacterial adhesion between azido‐modified bacteria (green) and DBCO‐modified bacteria (red). Scale bars: 1 µm. Reproduced under terms of the CC‐BY license.^122^ Copyright 2022, The Authors, published by American Chemical Society.

The strong interaction between probiotics and the intestinal mucus layer is crucial for their colonization in the gut. By increasing the adhesion of probiotics to the intestinal mucus layer, their intestinal retention capacity can be significantly improved, thereby promoting their final therapeutic efficiency. In recent years, many probiotic adhesion strategies have been proposed to this end.^119^ For example, inspired by adhesins that promote bacterial colonization, Anselmo and colleagues attached streptomycin‐conjugated anti‐MUC2 antibody on biotinylated EcN to achieve targeted adhesion, which significantly increased the colonization efficiency of EcN and prolonged their colonization time (Figure 6A,B).^120^ Shapiro and colleagues modified magnetic nanoparticles on the surface of probiotics using the biotin‐streptavidin method and enhanced the localization and colonization of oral probiotics with the aid of micromagnet amplifiers and an external magnetic field (Figure 6C,D).^121^ Zhang and colleagues developed a bioorthogonal conjugation strategy for probiotic delivery. Specifically, the peptidoglycan of gut bacteria was modified with azido‐modified D‐alanine, which in situ conjugated with dibenzocyclooctyne (DBCO)‐modified probiotics in complex biological environments (Figure 6E,F). In this way, the modified gut inhabitants served as artificial reaction sites, which enhanced probiotics retention and colonization.^122^

**FIGURE 7:**
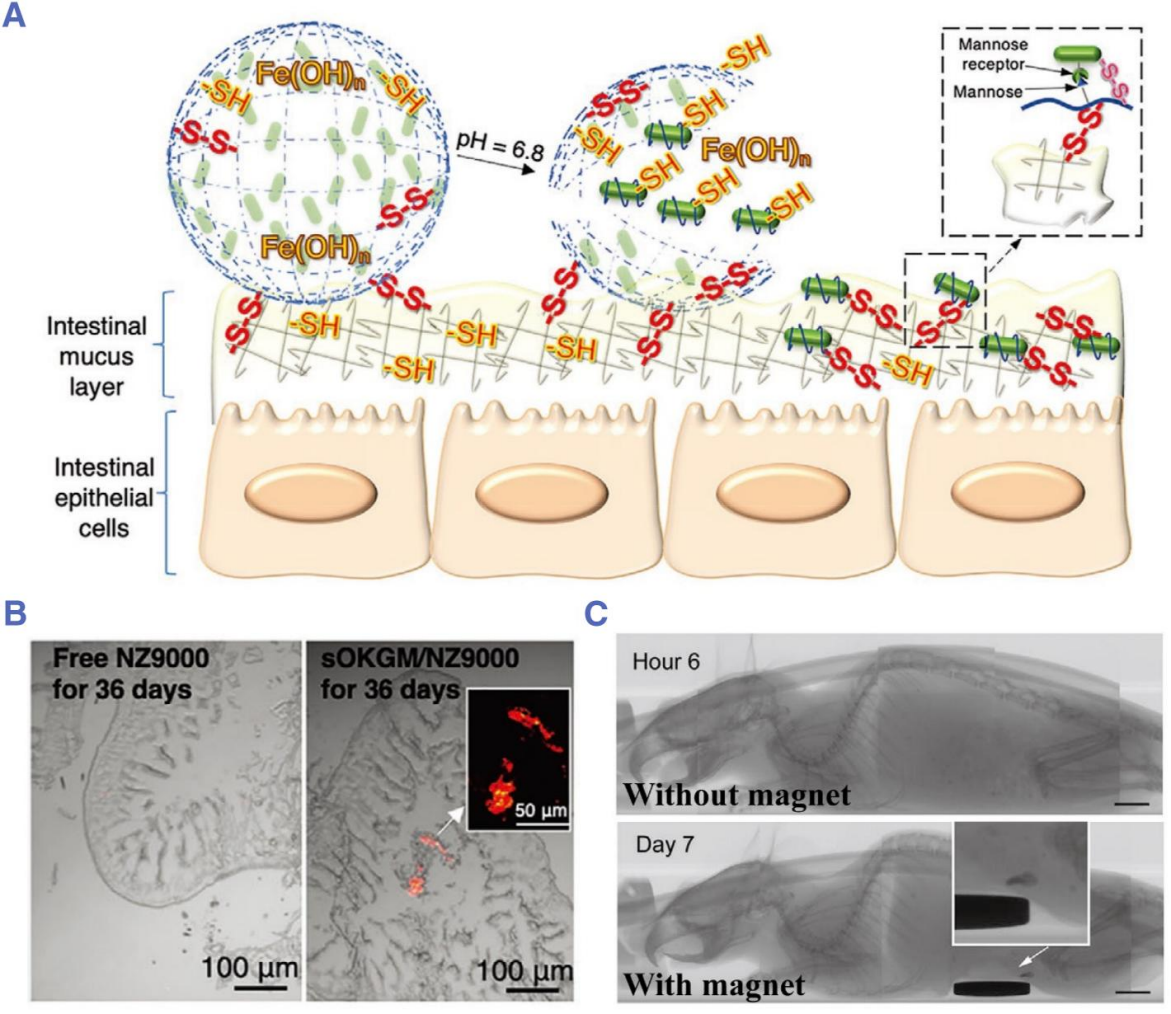
(A) Schematic showing thiolated oxidized konjac glucomannan used as a bridge to improve the mucosal adhesion of probiotics by forming disulfide bonds with mucus and probiotics. (B) Long‐term intestinal retention of free NZ9000 (red) and encapsulated NZ9000, NZ9000 refers to *Lactococcus lactis* NZ9000. Reproduced with permission.^34^ Copyright 2020, John Wiley and Sons. (C) The ingested magnetic live hydrogel containing probiotics passed through the whole gastrointestinal tract in 6 h without a magnet, while it remained in the intestine for 7 days in the presence of a magnet. Scale bars: 5 mm. Reproduced with permission.^128^ Copyright 2021, John Wiley and Sons.

In addition to surface modification of probiotics, encapsulation of probiotics within adhesive materials can also endow them with excellent adhesion properties.^35^ ^,^ ^123^, ^124^, ^125^ As a natural positively charged polysaccharide, chitosan has been used as an excipient for colon‐targeted drug delivery due to its colonic degradation properties and strong adhesion to mucosal tissues.^126^ ^,^ ^127^ Besides, Li and colleagues encapsulated probiotics into thiolated oxidized konjac glucomannan microspheres to improve their adhesive colonization ability.^34^ The polymer can form disulfide bonds with the probiotics and the mucus layer, allowing the probiotics to stay in the body for 36 days (Figure 7A,B). Alternatively, Wang and colleagues developed an ingestible magnetic hydrogel carrier containing magnetized neodymium‐iron‐boron (NdFeB) microparticles to embed genetically engineered probiotics EcN.^128^ The carrier achieved localization and long‐term retention of probiotics through the use of a wearable magnet, as illustrated in Figure 7C.

### 4.4 Mediating targeting and release

**FIGURE 8:**
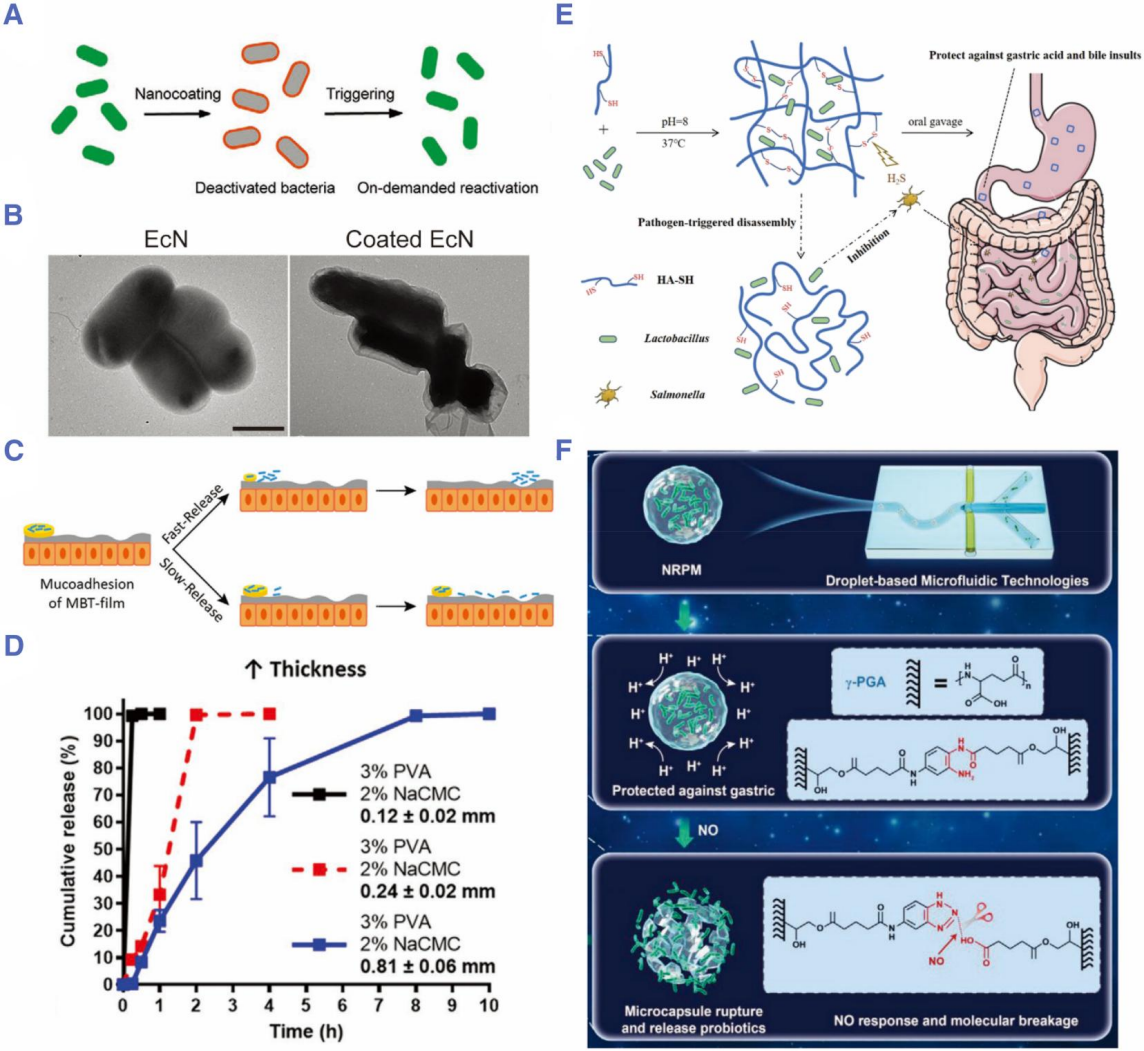
(A) Schematic of the pH‐triggered on‐demand bacterial activation process. (B) Transmission electron microscope (TEM) images of uncoated and coated EcN. Scale bar: 1 µm. Reproduced with permission.^130^ Copyright 2020, John Wiley and Sons. (C) Microbe‐based therapeutics (MBT) films designed for tunable release of probiotics along the intestines. (D) Kinetic curve of probiotic release under various film thicknesses. Reproduced with permission.^131^ Copyright 2020, John Wiley and Sons. (E) Schematic diagram of HA‐based self‐crosslinking hydrogel for pathogen‐targeted delivery and responsive release of *Lactobacillus*. Reproduced with permission.^38^ Copyright 2020, American Chemical Society. (F) Schematic diagram of the fabrication process and working principle of intestinal‐targeted NO‐responsive hydrogel microcapsules. Reproduced with permission.^132^ Copyright 2022, John Wiley and Sons.

Apart from surviving the harsh environment of the gastrointestinal tract, probiotics need to be targeted to the intestinal tract and released to fully exert their efficacy. In view of the unique pH characteristics of the gastrointestinal tract, pH‐sensitive probiotic delivery systems have been developed. For example, alginate‐based pH‐responsive microspheres have been prepared to encapsulate probiotics, which enabled targeted release in the small intestine.^129^ Liu and colleagues used enteric coating (Eudragit L100‐55) to make probiotics inactive in the stomach. After reaching the intestine, the probiotics were reactivated by pH‐triggered decoating, thus exerting their effects (Figure 8A,B).^130^ Another method for precise targeted delivery and release relies on the use of antibodies. Wang and colleagues fabricated alginate microspheres with a chitosan shell encapsulating *Lactococcus lactis.* By adding small intestinal targeting antibody‐proton‐dependent transporter 1 to the chitosan shell, the system facilitated targeted delivery and spatiotemporal manipulation of orally delivered *Lactococcus lactis*.^133^ In addition, the release efficiency of probiotics can be altered by adjusting the thickness of the coating. Anselmo and colleagues found that increasing the thickness of a polymer film composed by poly(vinyl alcohol) (PVA) and sodium carboxymethyl cellulose (NaCMC) could significantly prolong the release curve of *L. caceiat* CC393 (Figure 8C,D).^131^

In addition to designing targeted release strategies according to the physiological characteristics of the gastrointestinal tract, researchers have also been focused on the intestinal pathological environment.^134^ For example, under salmonellosis conditions, the intestinal pathogens *Salmonella* excrete a large amount of H_2_S.^135^ Based on this, Duan and colleagues formed hydrogels to encapsulate probiotics by self‐crosslinking of thiol‐modified hyaluronic acid (HA) (Figure 8E).^38^ After exposure to H_2_S, the disulfide bond was broken, and the hydrogel could degrade locally and release the probiotics quickly. Xu and colleagues proposed nitric oxide (NO)‐responsive poly‐γ‐glutamate (γ‐PGA) hydrogel microcapsules based on droplet microfluidics.^132^ The microspheres can rapidly release probiotics in response to NO stimulation (Figure 8F). These efforts indicate that, combining relevant characteristics of the physiological or pathological environments with responsive materials, smart delivery systems can be developed to achieve probiotics targeting and controlled release.

### 4.5 Regulation of pathological microenvironment

**FIGURE 9:**
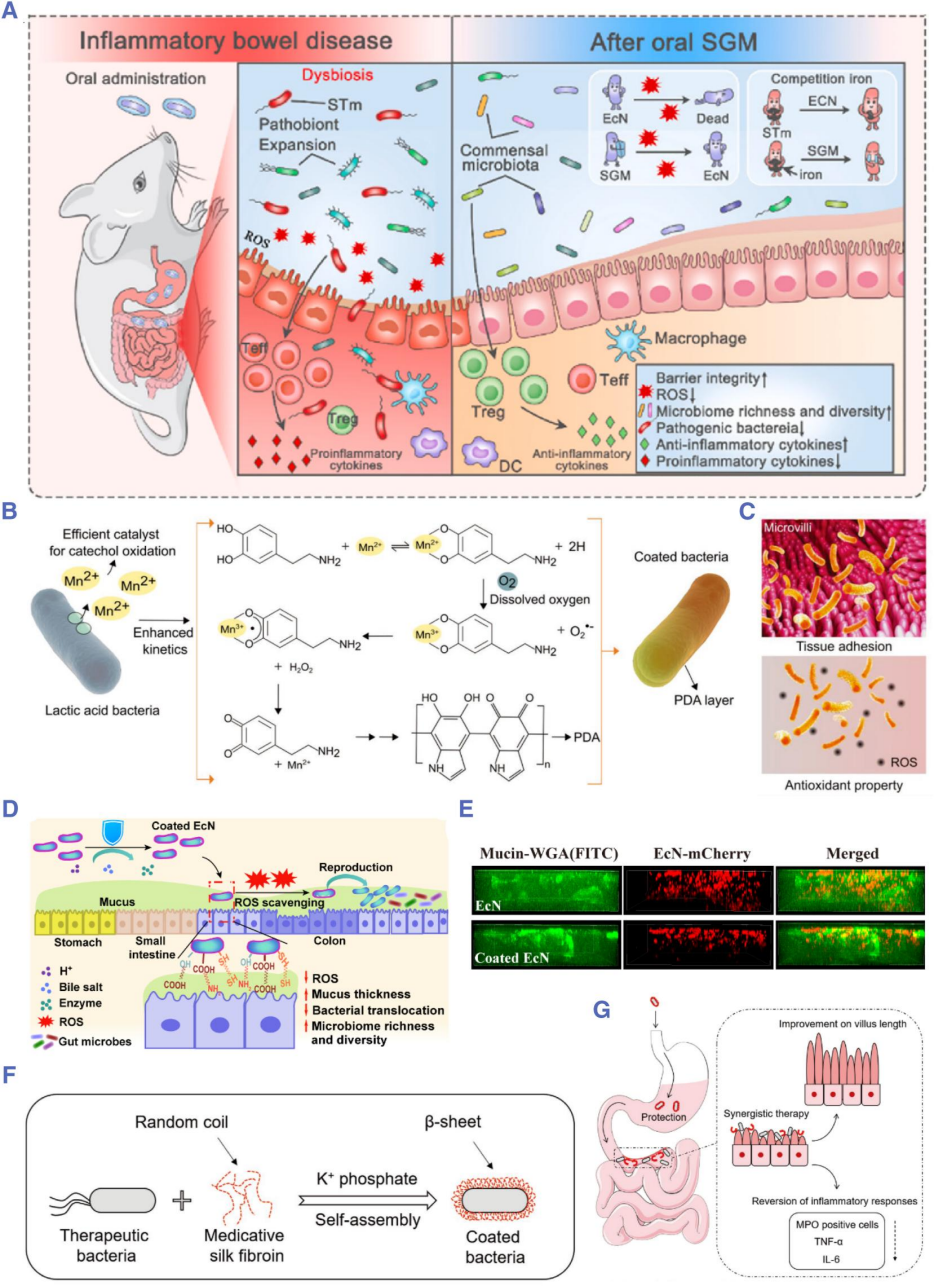
(A) Schematic diagram of the nanocoating system for scavenging reactive oxygen species (ROS), inhibiting pathogenic bacteria, and promoting intestinal homeostasis after oral administration. Reproduced with permission.^40^ Copyright 2022, Elsevier. (B) Probiotic‐mediated catechol compounds oxidation and their in situ nanocoating formation. (C) The enhanced mucoadhesion and antioxidant properties of the phenolic oxidation nanocoating. Reproduced under terms of the CC‐BY license.^138^ Copyright 2022, The Authors, published by John Wiley and Sons. (D) Enhanced intestinal colonization with the use of mucin‐based nanocoating on EcN, which modulates pathological microenvironment. (E) Images showing mucus penetration of uncoated and coated probiotics. Reproduced with permission.^24^ Copyright 2022, American Chemical Society. (F) Decorating probiotics with a silk fibroin nanocoating through biointerfacial self‐assembly. (G) Synergistic therapeutic effect rendered by the nanocoating. Reproduced with permission.^139^ Copyright 2021, John Wiley and Sons.

In addition to the normal intestinal physiological environment, oral probiotics also encounter pathological factors under disease conditions, such as elevated ROS, depleted mucus layer, and increased pathogenic bacteria, as mentioned above. With the development of oral probiotic delivery systems, the use of biomaterials in synergy with probiotics has gained increasing attention for the enhancement of biotherapeutics efficacy through modulation of the pathological status of diseases. As a polyphenol, tannic acid is rich in pyrogallol groups and can effectively scavenge ROS.^136^ In addition, it can rapidly coordinate with metallic ions to form complexes.^137^ In view of this, Shi and colleagues used tannic acid and poloxamer 188 to form a self‐assembled nano‐coating on the surface of probiotics.^40^ The armor can significantly reduce intestinal inflammation, and it competitively competes with intestinal pathogens for iron ions on which they depend for survival, thereby suppressing pathogens' colonization and modulating the intestinal flora composition (Figure 9A). In addition, polydopamine is often used as a ROS scavenger since it contains catechol groups.^140^ ^,^ ^141^ Rahim and colleagues reported a nanocoating formed by Mn ion‐mediated oxidative self‐polymerization of dopamine on the surface of probiotics, which showed potent radical scavenging ability and mucosal adhesion capacity (Figure 9B,C).^138^

Since probiotics colonize and grow in the mucus layer, Shi and colleagues used mucin, a major component of mucus, to modify probiotics, which increased the viscoelasticity and barrier function of the damaged mucus layer through strong interactions of homologous proteins.^24^ The mucin coating also reduced the penetration of probiotics in the mucus layer and reduced the inflammatory side effects of bacterial translocation, thus fully utilizing the therapeutic efficacy of probiotics (Figure 9D,E). As a common drug delivery vehicle, silk fibroin‐based materials also have their own anti‐inflammatory properties.^142^ Based on this, Liu and colleagues used silk fibroins to form nanocoating on the surface of probiotics through self‐assembly, which not only provided barrier protection but also reduced inflammation and synergistically enhanced probiotic therapy (Figure 9F,G).^139^ In addition, catalytic nanomaterials with enzymatic activity (referred to as nano‐enzymes) are emerging and promising antioxidants that are commonly used for the treatment of inflammation associated with ROS.^143^ ^,^ ^144^ Ma and colleagues demonstrated that the incorporation of nano‐enzymes‐based coating materials on probiotics also contributed to reduced inflammatory effects and tissue damage.^145^

## 5 ORALLY DELIVERED ARMORED PROBIOTICS FOR DISEASE TREATMENT

Oral probiotics, as an active microbial supplement, have intrinsic therapeutic effects. However, the physiological and pathological environment of the gastrointestinal tract greatly limits the bioavailability of probiotics. Strategies for decorating or encapsulating probiotics with the use of a variety of functional materials have been developed for disease treatment to address the above challenges.^146^ The resultant probiotic armors can not only improve the survival and colonization of orally delivered probiotics in the intestinal tract but also integrate additional functions, thus enhancing the therapeutic performances of probiotics for both intestinal‐related diseases and other diseases.

### 5.1 IBD

**FIGURE 10:**
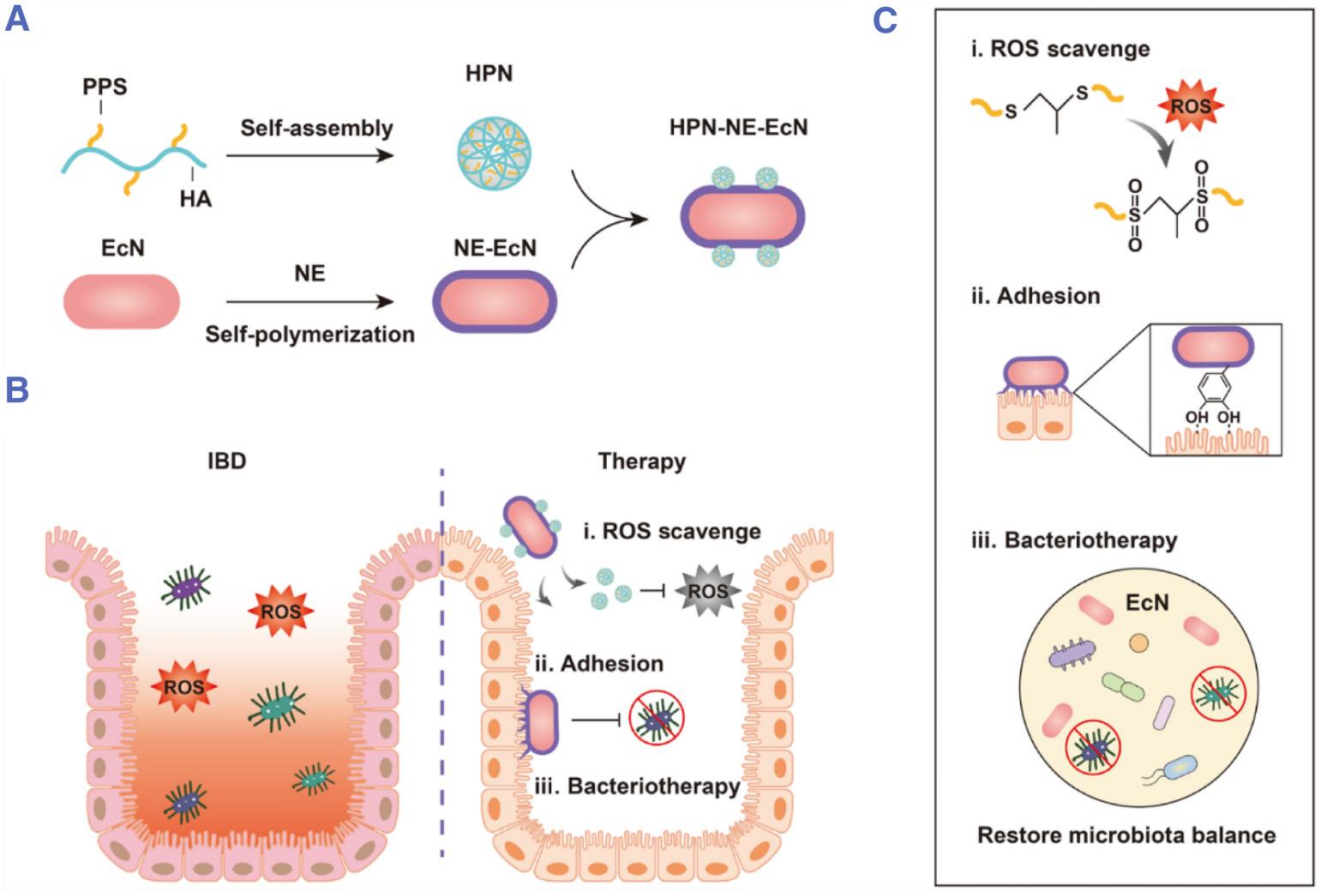
(A) HA‐PPS nanoparticles (HPN) were coupled to EcN with a norepinephrine coating. (B, C) The coated probiotics (i) exert reactive oxygen species (ROS) scavenging ability through PPS reactivity, (ii) extend intestinal retention time through mucosal adhesion of the norepinephrine layer, and (iii) enhance probiotics' therapy performance by restoring intestinal homeostasis. Reproduced with permission.^152^ Copyright 2022, The Authors, published by American Association for the Advancement of Science.

Intestinal flora plays an important role in regulating human intestinal homeostasis. Studies have shown that intestinal flora imbalance is associated with a variety of gastrointestinal diseases, such as IBD,^147^ ^,^ ^148^ colorectal cancer^149^ and so on. IBD can be triggered by genetic predisposition, immune mechanisms, and environmental factors.^150^ One of the important biochemical indicators of IBD is the significantly elevated ROS concentration in the intestinal mucosa, which can also aggravate the occurrence and development of IBD by inducing oxidative stress.^151^ After oral administration, armored probiotics can exert a good therapeutic effect on IBD. For example, Hu and colleagues modified probiotic EcN with a polynorepinephrine coating and equipped the surface of the coating with HA‐poly (propylene sulfide) (HA‐PPS) nanoparticles (Figure 10A).^152^ With that, PPS can effectively eliminate ROS, and the norepinephrine coating extended the retention time of the therapeutic probiotics in the intestinal tract. This enhanced the efficacy of probiotics in alleviating dextran sulfate sodium (DSS)‐induced colitis in mice (Figure 10B,C).

### 5.2 Colorectal cancer

**FIGURE 11:**
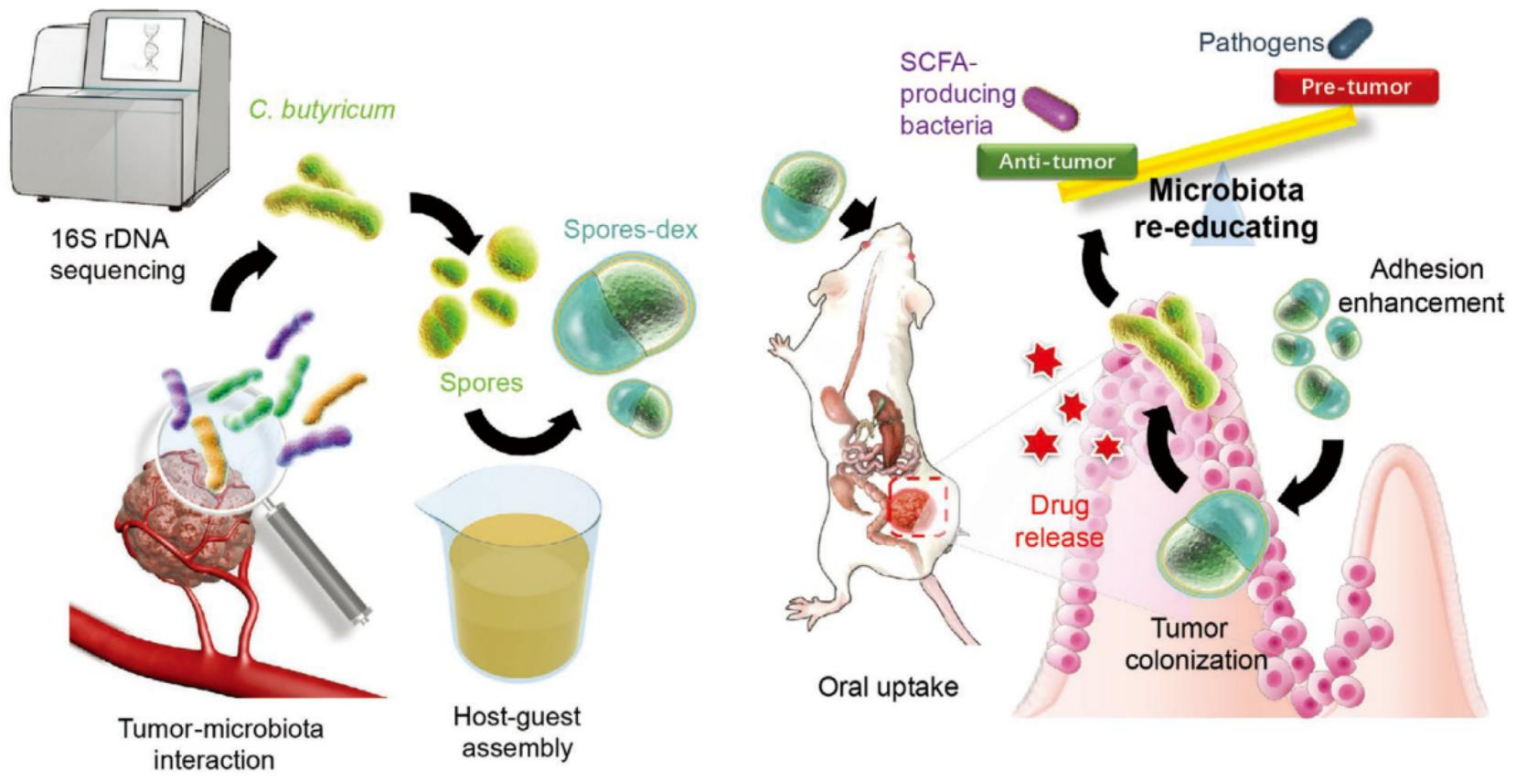
Schematic illustration of the dextran‐coated probiotic spores regulating the gut microbiota and inhibiting colon cancer. Reproduced with permission.^37^ Copyright 2020, John Wiley and Sons.

Colorectal cancer is closely associated with the ecological imbalance of intestinal microbiota in colonic microenvironment.^153^ The intervention of probiotics also has the ability to regulate and treat colorectal cancer, and the hypoxic tumor microenvironment enables anaerobic therapeutic bacteria tend to be efficiently enriched in the lesions.^154^ Probiotics can suppress tumors by competing with pathogenic bacteria for nutrients, modulating metabolism, and activating immunity.^155^ Zhang and colleagues carried out an in‐depth analysis of the microbial flora composition of the colon tumor site and selected probiotics *Clostridium butyricum* for specific targeting.^37^ After oral administration, the anaerobic *Clostridium butyricum* spores, coated with prebiotic dextran, were specifically enriched in colon cancer tissues. Through germination, dextran was metabolically decomposed by *Clostridium butyricum*, which significantly stimulated the secretion of anticancer metabolites. This contributed to a synergetic effect with the probiotics for the treatment of colon cancer (Figure 11).

### 5.3 Other diseases

In addition to intestinal diseases, armored probiotics also have a good therapeutic effect on metabolic syndrome, alcoholic liver disease, and other diseases. For distal disease sites where probiotics cannot reach, probiotics can function by regulating the intestinal flora. Specifically, gut microbes can produce some beneficial metabolites, such as trimethylamine‐N‐oxide, SCFAs, and secondary bile acids, which affect the pathogenesis of cardiovascular diseases. In addition, alterations in gut microbiota composition are associated with endothelial dysfunction, inflammation, and oxidative stress, which cause potential risk of developing metabolic diseases.^146^ Shang and colleagues fabricated microcapsules with a shell of prebiotics to wrap two kinds of probiotics, which not only protected the activity of the probiotics but also enhanced their synergism. The proposed microcapsules demonstrated abilities in alleviating fat deposition in liver and relieving intestinal inflammation, thus showing potential for the treatment of high fat diet‐induced metabolic syndrome.^118^ In addition, Kaur and colleagues co‐encapsulated prebiotics epigallocatechin gallate (EGCG) and probiotics in calcium alginate microbeads.^156^ The microbeads promoted effectively regulated the levels of alcohol, endotoxin and liver enzymes in the body. Such a combination system is expected to become a treatment choice for the management of alcoholic liver disease. Overall, it is an attractive way to improve the therapeutic effect of probiotics by modifying them with various functional materials for different purposes. The decorative materials with good biocompatibility and functionalities provide a unique perspective for obtaining better therapeutic outcomes, and more biomedical and clinical applications can be explored in the future.

## 6 CONCLUSION AND PERSPECTIVE

In recent decades, the cross‐fusion of material science and microbiological science has greatly promoted research on oral probiotic delivery, and the development of functional material‐armored probiotics has made remarkable progress. This paper systematically summarizes how different armor materials endow probiotics with different functions to help maximize their efficacy and the applications of armored probiotics in disease treatment. With the discovery of new materials and new functions of materials, researchers have made a lot of explorations to solve the difficulties encountered in oral probiotic delivery. For example, arming probiotics with some acid‐resistant materials can effectively withstand the harsh physiological environment of the gastrointestinal tract; biomaterials with prebiotic properties can promote the vitality of probiotics; adhesive materials that encapsulate or decorate probiotics can significantly increase the retention capacity of probiotics in the intestinal tract. Biomaterials with pH, enzyme, light, or magnetic responsiveness can promote the targeted delivery of probiotics in the intestinal tract, and those with disease regulation abilities (such as anti‐inflammatory and ROS scavenging properties) can enhance the therapeutic ability of probiotics. In general, the principle for armored probiotics is to not only maintain the natural activity and function of probiotics but also give probiotics exogenous functions, so as to achieve greater therapeutic performances. In addition, it is found that not only the natural physiological barrier of the gastrointestinal tract is to be taken into account, more importantly, the changes of the intestinal microenvironment under disease conditions also highlight the necessity of the use of functional armor materials in mediating the probiotic delivery process.

Oral probiotics delivery represents an emerging trend in “living therapy”. Although huge progress has been achieved, and various experimental models have been established, there still is a long way to go before they are widely used in the clinic. The safety, effectiveness, and accuracy of clinical translation of probiotic therapy remain to be solved. Nevertheless, it is foreseeable that the use of clinically approved functional biomaterials can assist in addressing these challenges. Future endeavors may be focused on reducing the immunogenicity and at the same time, endowing probiotics with novel functions. Besides, the decoration or encapsulation strategies of probiotics can further benefit from advanced processing techniques such as microfluidics and three‐dimensional printing, by which the structure, physicochemical features, and biological activities of the armored probiotics can be finely regulated. Moreover, combining probiotics with other therapeutic regimes, such as drug therapy, may bring new solutions to disease treatment. To sum up, we believe that with the continuous expansion of new materials and material processing methods, the therapeutic effect of probiotics can be optimized and the application prospect of probiotic delivery can be enriched.

## AUTHOR CONTRIBUTIONS

Luoran Shang and Xinyuan Yang conceived the idea; Xinyuan Yang and Luoran Shang wrote the original manuscript; Luoran Shang, Chong Wang, Qiao Wang, Zhuohao Zhang and Weimin Nie revised the manuscript.

## CONFLICT OF INTEREST STATEMENT

Luoran Shang is an executive editor for *Smart Medicine* and was not involved in the editorial review or the decision to publish this article. All authors declare that there are no competing interests.

## ETHICS STATEMENT

This paper does not cover studies in human subjects or animal studies.
